# Autonomic function may not modulate irisin release in healthy adults: findings from a randomized cross-over study

**DOI:** 10.20945/2359-3997000000243

**Published:** 2020-06-05

**Authors:** Shanhu Qiu, Edit Bosnyák, Martina Zügel, Jürgen Michael Steinacker, Uwe Schumann

**Affiliations:** 1 Shenzhen People’s Hospital Clinical Medical College Jinan University China Department of Endocrinology,Shenzhen People’s Hospital; The Second Clinical Medical College of Jinan University; The First Affiliated Hospital of Southern University of Science and Technology, Shenzhen, China; The First Affiliated Hospital Southern University of Science and Technology Shenzhen China; 2 Zhongda Hospital School of Medicine Southeast University Nanjing China Department of Endocrinology, Zhongda Hospital, Institute of Diabetes, School of Medicine, Southeast University, Nanjing, China; 3 Division of Sports and Rehabilitation Medicine Ulm University Medical Center Ulm Germany Division of Sports and Rehabilitation Medicine, Ulm University Medical Center, Ulm, Germany; 4 Department of Health Sciences and Sports Medicine University of Physical Education Budapest Hungary Department of Health Sciences and Sports Medicine, University of Physical Education, Budapest, Hungary

**Keywords:** Autonomic function, irisin, heart rate, exercise

## Abstract

**Objective:**

Autonomic nervous system, especially the sympathetic nervous system, may stimulate the expression of peroxisome proliferator-activated receptor γ coactivator-1α, which regulates irisin. This study aimed to explore whether there was any association between autonomic function as assessed by heart rate related indices and irisin release following acute exercise.

**Subjects and methods:**

Seventeen healthy adults were asked to perform an incremental exhaustive cycling as well as an incremental exhaustive running separately on different days. Heart rate was monitored, and blood samples were collected before, immediately, 10-, and 60-minutes post-exercise. Serum irisin was measured using ELISA kit.

**Results:**

Markers for autonomic function, such as heart rate at rest, peak, or recovery, heart rate reserve, heart rate recovery, and chronotropic index, were comparable between cycling and running (all P > 0.10). Irisin was increased immediately following both exercise. No significant association was observed between heart rate at rest, peak, or recovery and irisin level at the corresponding time-point, as well as between heart rate reserve, heart rate recovery, or chronotropic index and exercise induced irisin release, with or without controlling for age, body mass index, and glucose (all P > 0.10).

**Conclusions:**

Autonomic function might not be associated with irisin release in healthy adults. Arch Endocrinol Metab. 2020;64(3):201-4

## INTRODUCTION

As one of the two major components of the autonomic nervous system, the sympathetic nervous system plays an important role in modulating the metabolism of brown adipose tissue ( 1 ). Further evidence from the animal experiments suggests that the activation of sympathetic nervous system may also stimulate the peroxisome proliferator-activated receptor γ coactivator-1α (PGC-1α) expression ( 2 ). Since irisin, a newly identified hormone that drives the browning of white adipose tissue, was shown to be regulated by PGC-1α ( 3 ), it seems biologically plausible that the autonomic nervous system, in particular the sympathetic nervous system, may have a regulatory role pertaining to irisin. Yet this assumption has not been explored in adult humans.

Heart rate is a simple and noninvasive indicator of autonomic function and reflects the coordinated interplay between sympathetic and parasympathetic tone ( 4 , 5 ). It is increased markedly during exercise mainly because of sympathetic activation and is decreased gradually post-exercise due to parasympathetic activation and sympathetic withdrawal, especially in the late recovery period (e.g., 5-min post exercise) ( 5 , 6 ). Given these and considering that irisin shows a similar pattern – that is, irisin is transiently increased following acute exercise ( 7 ), this study was aimed to examine whether there exists any association between autonomic function as represented by heart rate related indices and irisin release throughout the exercise and recovery periods.

## SUBJECTS AND METHODS

Healthy nonobese individuals aged less than 40 years were recruited via notices at the Division of Sports and Rehabilitation Medicine, Ulm University Medical Center, Ulm University. This study complies with the standards set by the Declaration of Helsinki and was approved by the Research Ethics Committees of Ulm University. Written informed consent was obtained from all subjects.

The protocol of this study has been described previously ( 8 ). In brief, 17 subjects (8 men and 9 women, mean age: 26.8 ± 5.7 years, mean body mass index: 23.9 ± 2.3 kg/m^2^) performed 2 different bouts of acute exercise (that is, incremental exhaustive cycling and running) on separate days with an interval of 1-week using a randomized cross-over design. All subjects were asked to have a rest following these 2 bouts of acute exercise for recovery for 60 minutes. Heart rate was monitored throughout this time-window using a wireless heart rate monitor (CardioPart 12 Blue, Amedtec, Aue, Germany). Blood samples were drawn before, immediately, 10-, and 60-min after the cessation of each exercise. Heart rate reserve was defined as the difference between the heart rates at peak and rest ( 9 ), whereas heart rate recovery was as the heart rate at peak minus the heart rate at 10 minutes of recovery after exercise in this study. Chronotropic index was calculated as heart rate reserve divided by (220 – age – heart rate at rest) ( 10 ). Serum irisin was measured using a commercial ELISA kit (Phoenix Pharmaceuticals, Burlingame, CA, USA; EK-067-52), and capillary glucose was determined by glucose oxidase method. Student *t* test was used to compare the differences between groups, and Pearson correlation analysis was applied to assess these associations. A 2-sided *P* value < 0.05 was considered statistically significant. All analyses were conducted using PASW 18.0 (SPSS, Inc., Chicago, Illinois).

## RESULTS

The heart rate at peak was achieved at the time-point of immediately post-cycling or -running. Although the time to exhaustion was longer for cycling than running (0.41 *versus* 0.31 hours, *P* = 0.003), the heart rate at rest, peak, or recovery, heart rate reserve, heart rate recovery, and the chronotropic index were comparable (all *P* > 0.10). Serum irisin was increased immediately following incremental exhaustive cycling or running but began to decline gradually later (data not shown).

**Figure 1:**
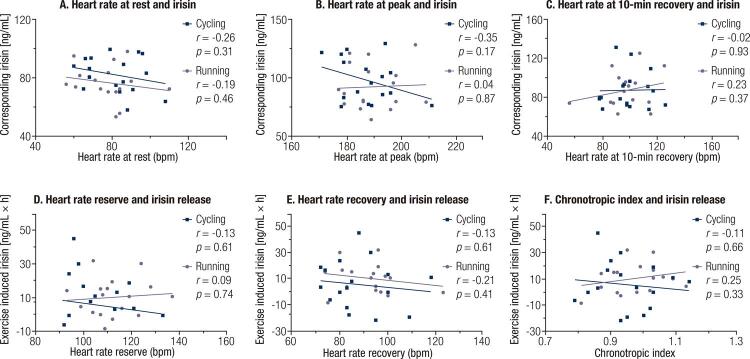
Association between markers of autonomic function with irisin release following 2 different protocols of acute exercise. (A) The association of heart rate at rest with corresponding irisin level; (B) The association of heart rate at peak with corresponding irisin level; (C) The association of heart rate at 10-min recovery with corresponding irisin level; (D) The association of heart rate reserve with exercise induced irisin release; (E) The association of heart rate recovery with exercise induced irisin release; (F) The association of chronotropic index with exercise induced irisin release.

**Figure 2:**
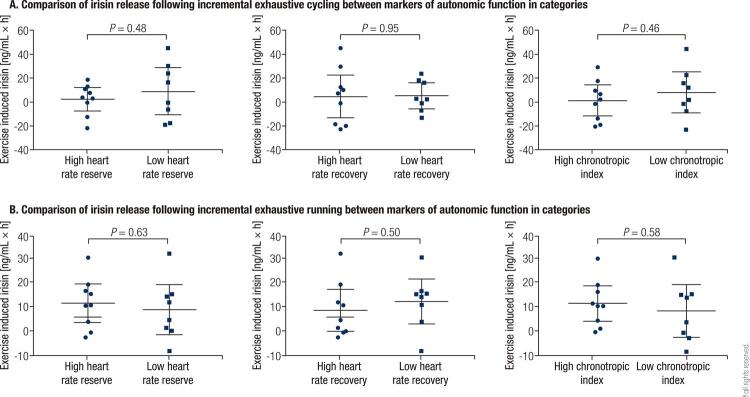
Comparison of irisin release following acute exercise between markers of autonomic function in categories.Data were expressed as means and their 95% confidence intervals. (A) The comparison of irisin release following incremental exhaustive cycling between markers of autonomic function in categories; (B) The comparison of irisin release following incremental exhaustive running between markers of autonomic function in categories.

Correlation analyses suggested that no association was observed between heart rate at rest, peak, or recovery and irisin level at the corresponding time-point (all *P* > 0.10, Figures 1A, B, and C ), even after controlling for age, body mass index, and capillary glucose at rest (all *P* > 0.10). In addition, exercise induced irisin release throughout the exercise and 60 minutes recovery periods, which was represented as the area under the curve that is calculated using the trapezoidal method, showed no significant association with heart rate reserve, heart rate recovery, or chronotropic index (all *P* > 0.30, Figures 1D, E, and F ). These associations remained still non-significant after adjusting for age, body mass index, and capillary glucose at rest (all *P* > 0.25). Moreover, after grouping heart rate reserve, heart rate recovery, and chronotropic index into the high (not below the median) and low (below the median) categories separately, no significant difference regarding exercise induced irisin release was noted across these categories (all *P* > 0.40, Figure 2 ).

## DISCUSSION

Our study suggested that none of the heart rate related indices in response to cycling or running was associated with the corresponding irisin level at rest, peak or recovery, or correlated with irisin release throughout the exercise and recovery periods, even after adjusting for age, body mass index, and glucose. These indicate that autonomic function might play a limited role in regulating irisin release.

Our results are in support of the findings from the previous report by Scalzo and cols. who observed that decreased sympathetic activity induced by clonidine and increased sympathetic activation mediated by hypoxia had little influence on circulating irisin ( 11 ). Yet our results did not correspond well with the hypothesis that the activation of sympathetic nervous system, a major component of the autonomic nervous system, may upregulate the expression of PGC-1α, and consequently induce irisin release. It is speculated that not all the stimulators, which could activate PGC-1α, are necessarily responsible for irisin release. Moreover, our study may also suggest that the reduced risk of diabetes related to lowered resting heart rate ( 4 ) or increased heart rate recovery ( 12 ) cannot be well explained by the lowered irisin levels that coincide with decreased risk of diabetes ( 13 ), albeit irisin is associated with improved metabolic profile and could ameliorate insulin resistance and increase glucose uptake in skeletal muscles ( 14 , 15 ).

Our study is the first report that examines the potential relationship of autonomic function as signified by heart rate related indices with irisin release following 2 different acute exercise protocols. However, the relatively small sample size might weaken the robustness of our findings, which may require further validation. Moreover, the limited sample size did not enable us to perform subgroup analyses on the basis of the baseline characteristics of enrolled individuals such as physical status that is considered to affect the autonomic function ( 5 ).

In conclusion, autonomic function seems to have no association with irisin release in healthy adults. Yet it requires to be confirmed by future studies having larger sample sizes conducted in healthy adults or among diseased individuals such as patients with polycystic ovary syndrome who showed insulin resistance and lower irisin levels than healthy controls ( 16 ). Moreover, using different approaches in addition to acute exercise to modulate autonomic function such as Valsalva Maneuver or deep breathing ( 17 ) may also provide some insights into the investigation of the association between autonomic function and irisin.
